# Hellenic registry of patients with home mechanical ventilation (HR-HMV): profiling sleep Apnea–Hypopnea syndrome patients across Greece

**DOI:** 10.1186/s12911-022-01989-1

**Published:** 2022-10-01

**Authors:** Vlasios K. Dimitriadis, Christina Alexopoulou, Anastasia Amfilochiou, Paschalis Steiropoulos, Georgia Trakada, Dimitra Siopi, Athanasia Pataka, Elpis Hatziagorou, Athanasios Konstandinidis, HR-HMV Data Curation Group, Georgia Varsou, Anastasia Chasiotou, Anastasios Kallianos, Athanasios Gounidis, Eumorfia Kondili, Charikleia Tselepi, Georgia Chasapidou, Athanasios Voulgaris, Kalamaras George, Elissavet-Anna Chrysochou, Michalis Agrafiotis, Maria Antoniadou, Porpodis Konstantinos, John Tsanakas, Venetia Tsara, Pantelis Natsiavas

**Affiliations:** 1 Institute of Applied Biosciences, Centre for Research and Technology Hellas, 6th Km. Charilaou – Thermi Road, Thermi, P.O. BOX 60361, 57001 Thessaloniki, Hellas Greece; 2 Intensive Care Unit and Sleep Lab, University Hospital of Heraklion, Heraklion, Greece; 3 Respiratory Function and Sleep Study Unit, Sismanoglio General Hospital of Attica, Athens, Greece; 4 Sleep Unit, Department of Pulmonology, Medical School, Democritus University of Thrace, Alexandroupolis, Greece; 5 Department of Clinical Therapeutics, Division of Pulmonology, National and Kapodistrian University of Athens, School of Medicine, Alexandra Hospital, Athens, Greece; 6 Pneumonology Clinic, G. Papanikolaou Hospital, Aristotle University of Thessaloniki, Thessaloniki, Greece; 7 Respiratory Failure Unit, G. Papanikolaou Hospital, Aristotle University of Thessaloniki, Thessaloniki, Greece; 8 Pediatric Pulmonology and Cystic Fibrosis Unit, Hippokration Hospital, Aristotle University of Thessaloniki, Thessaloniki, Greece; 9 Division of Respiratory Medicine, University Hospital of Ioannina, University of Ioannina Medical School, Ioannina, Greece; 10 Sleep Lab, “G. Papanikolaou” Hospital, Aristotle University of Thessaloniki, Thessaloniki, Greece; 11 Sleep Lab, Agios Pavlos Hospital, Thessaloniki, Greece; 12 Sleep Lab, University Hospital of Ioannina, University of Ioannina Medical School, Ioannina, Greece; 13 Pneumonology Department and Sleep Lab, Giannitsa Hospital, Giannitsa, Greece

**Keywords:** Patient registries, Home mechanical ventilation, Chronic respiratory failure, Sleep Apnea–Hypopnea syndrome (SAHS)

## Abstract

**Background:**

Chronic respiratory conditions are a prominent public health issue and thus, building a patient registry might facilitate both policy decision making and improvement of clinical management processes. Hellenic Registry of patients with Home Mechanical Ventilation (HR-HMV) was initiated in 2017 and a web-based platform is used to support patient data collection. Eighteen hospital departments (including sleep labs) across Greece participate in this initiative, focusing on recording data for both children and adult patients supported by mechanical ventilation at home, including patients with Sleep Apnea–Hypopnea Syndrome (SAHS) under Positive Airway Pressure (PAP) therapy.

**Methods:**

The HR-HMV initiative ultimately aims to provide a database for evidence-based care and policy making in this specific domain. To this end, a web information system was developed and data were manually collected by clinics and hospital departments. Legal and privacy issues (such as General Data Protection Rule compliance and technical information security measures) have been considered while designing the web application. Based on the collected data, an exploratory statistical report of SAHS patients in Greece is presented.

**Results:**

Eleven out of the eighteen participating clinics and hospital departments have contributed with data by the time of the current study. More than 5000 adult and children patient records have been collected so far, the vast majority of which (i.e., 4900 patients) diagnosed with SAHS.

**Conclusion:**

The development and maintenance of patient registries is a valuable tool for policy decision making, observational/epidemiological research and beyond (e.g., health technology assessment procedures). However, as all data collection and processing approaches, registries are also related with potential biases. Along these lines, strengths and limitations must be considered when interpreting the collected data, and continuous validation of the collected clinical data per se should be emphasized. Especially for Greece, where the lack of national registries is eminent, we argue that HR-HMV could be a useful tool for the development and the update of related policies regarding the healthcare services for patients with home mechanical ventilation support and SAHS patients, which could be useful for related initiatives at a European level as well.

## Background

Chronic Respiratory Diseases (CRDs) are causing significant social and financial burden [1] and constitute one of the leading causes of mortality and disability worldwide, killing more than 3 million people every year. According to the Institute for Health Metrics and Evaluation (IHME)^1^ data for 2019, the prevalent cases of CRDs were around 454 million, while incident cases were more than 77 million. Moreover, CRDs were also accounted for more than 99 million disability-adjusted life years^2^. In Europe, until 2017, 15% of deaths were estimated due to respiratory diseases, with 600,000 people dying every year [2].

According to the European Respiratory Roadmap [3], research infrastructures such as databases, and nation-wide or EU-wide disease registries could play an important role in identifying population-level characteristics and facilitate policy decision making. Furthermore, as they could also support observational research, they could provide useful insights to support clinical management and thus facilitate clinical practice guidelines. Thus, registries have been identified as a priority in terms of policy guidelines and financial support [4] as their contribution of registries is very significant in terms of patients monitoring for public health purposes. The data collected in such a system, can provide healthcare professionals with fruitful insights about the prevalence of a disease or potential gaps regarding healthcare services that could be improved, and help them ensure the safe usage and the effectiveness of drugs or medical devices under real-world conditions [1, 5]. All these are critical in order to speed up and enhance the credibility of relevant studies, as well as for the reduction of costs regarding mechanical ventilation and chronic respiratory failure treatment [1, 5].

Greece is a country with about 11 M habitants. In terms of patients with respiratory conditions, chronic obstructive pulmonary disease (COPD), was the fifth most common cause of death for 2019^3^. More specifically, 11.74% (1,158,106.91) of total prevalent cases concerned CRDs, while 4.97% (6,000.32) of total deaths from all causes were due to CRDs. Regarding prevalence of Obstructive Sleep Apnea per se, it has been estimated in 2012 to affect 28.6% of adults between 30 and 69 years old [6].

In terms of eHealth, despite some recent developments, Greece lacks robust nation-wide infrastructures. The most important Information Technology (IT) infrastructure is the e-Prescription system, the use of which is mandatory and as such it could be considered a valuable resource for population level research. However, since the e-Prescription system was built mostly aiming to support administrative purposes (e.g. the reduction of the financial burden of medicine prescription), it lacks important features in terms of supporting public health policy research and decision-making. Even worse, as no nation-wide Electronic Health Record (EHR) system/framework exists, data science aiming to support public health policy making lacks significant data sources.

The Hellenic Registry of patients with Home Mechanical Ventilation support (HR-HMV) was initiated in 2017, aimed to form a forum among the respective clinical units across Greece, while also providing the technical infrastructure to support the registry data collection and analysis. The ultimate goal is to support policy making and the improvement of patient management in Greece and beyond. This paper outlines the processes applied in the HR-HMV, the respective technical platform and also provides a profile of the Sleep Apnea–Hypopnea Syndrome (SAHS) patients in Greece, the most prevalent patient group identified in HR-HMV so far.

## Methods

### Objective and design

The HR-HMV web platform is hosting the Greek registry for patients using home mechanical ventilation, typically due to chronic respiratory or neurological diseases, also including children. All data are stored in a central database, available to the contributing multiple health centers and institutions across the country, mainly for research purposes^4^. The HR-HMV IT platform hosted by the Institute of Applied Biosciences at the Centre for Research and Technology Hellas (INAB|CERTH) is the advanced version of an older IT system [7]. The new system, operational since 2019, was adapted closer to the requirements of clinicians and everyday practice, capable of storing information for more clinical procedures and storing a larger amount of patient data, while being more secure and user-friendly at the same time.

HR-HMV registry complies with General Data Protection Regulation and also conforms to the national Greek and European legislation, by applying all the necessary security, consent and confidentiality policies to guarantee data privacy and fulfill ethical responsibility. As the infrastructure of the hosting institute (INAB|CERTH) is certified for information security (ISO 27000), data validation and encryption mechanisms have been deployed to guarantee information security.

Along these lines, each participating clinical site and INAB|CERTH have signed a memorandum of cooperation to legally bind against the required technical measures and ensure the appropriate framework of partnership and cooperation. Moreover, all registered patients were provided written information about the research and gave written informed consent to participate, as well as to having anonymized/pseudonymized data shared. For children under 18 years of age, consent was taken from their parent/legal guardian in addition to the consent from the child participant. Furthermore, all staff participants have signed a non-disclosure agreement, to ensure data protection, confidentiality and security policy acceptance.

To date, eighteen hospital departments from many parts of Greece have joined the HR-HMV network, most of which (eleven) have already contributed with a significant amount of data for patients prescribed with mechanical ventilation system to use at home. At the time of writing, more than 5000 patients were registered in the HR-HMV platform, via hospital departments covering six different districts of the country (Athens, Thessaloniki, Ioannina, Giannitsa, Alexandroupoli, Heraklion).

### User interface design

Data are collected by the clinical staff via a web user interface, specifically designed and developed to facilitate the data entry process. To this end, one of the key end user requirements was to enable both prospective and retrospective data entry and potential corrections in order to maximize data collected while also enabling potential corrections.

The design of the user interface was conducted in an iterative fashion, based on the requirements posed by clinical staff. More specifically, a group of physicians with clinical background (led by the author VT) were actively engaged in the detailed design of the user interface components (e.g., which fields of data are going to be stored, how they are going to be organized in web forms, how detailed should the data entry validation rules be etc.). This process also included comments and remarks regarding the system usability which were used as a beacon during the UI design and the overall platform development process.

### Data collection and storage

Data are collected by clinical personnel and stored applying strict protection and security policies, such as data anonymization/pseudonymization, authentication and authorization, consistent data export, backup and restoration mechanisms and infrastructures, as well as sensitive data (i.e. passwords) hashing, and input validation during data entry. Guidelines emerging from the aforementioned policies have been additionally communicated to the participating staff through the HR-HMV manual and a medical acronyms dictionary available to them, the demo presentations and the regular members’ contact via the HR-HMV mailing-list and teleconferences. The main purpose of all those actions and practices, was to fulfill to the utmost, data quality assurance and completeness.

### Statistical analysis

Statistical analysis was performed on the (so-far) collected data regarding Sleep Apnea–Hypopnea Syndrome (SAHS) patients, as they are the dominating patient group in the registry. Initially, apart from the validation requirements and mechanisms used during data entry, additional case conditions and criteria specific to each field or collection of fields were defined, to ensure that inconsistencies or systematic errors during data entry will be timely identified. Data cleansing and other quality control activities were applied to guarantee data consistency, completeness and the integrity of the data management.

More specifically, the following features/data are elaborated:

- Demographic features such as gender and age
- Background information such as education level and occupation
- Clinical and lifestyle features such as Body Mass Index (BMI), smoking habits, underlying medical conditions and comorbidities
- Respiratory system function test results (e.g., Arterial Blood Gases, spirometry, Maximum Inspiratory Pressure, Maximum Expiratory Pressure, Peak Cough Flow etc.)
- Sleep tests (polysomnography, level III tests, oximetry), and indexes such as Apnea Hypopnea Index/Respiratory Disturbance Index (AHI/RDI), Average Oxygen Saturation (avSaO2), Minimum Oxygen Saturation (MinSaO2) and Time needed to reach oxygen saturation below 90% (T90)
- Mechanical ventilation device used related information (e.g., type of device)
- Follow up details (e.g., compliance, family support, quality of life etc.)

## Results

### The HR-HMV platform

**Fig. 1:**
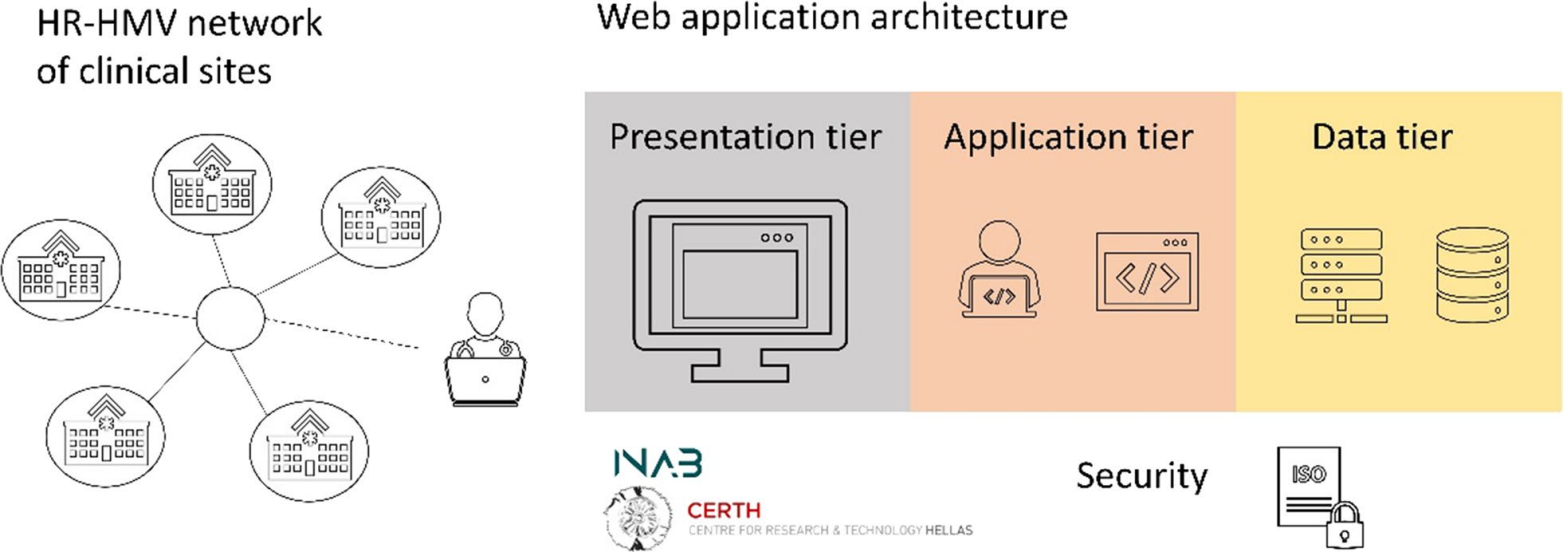
The HR-HMV platform architecture

The architecture of the HR-HMV platform is depicted in Fig. 1.

**Fig. 2:**
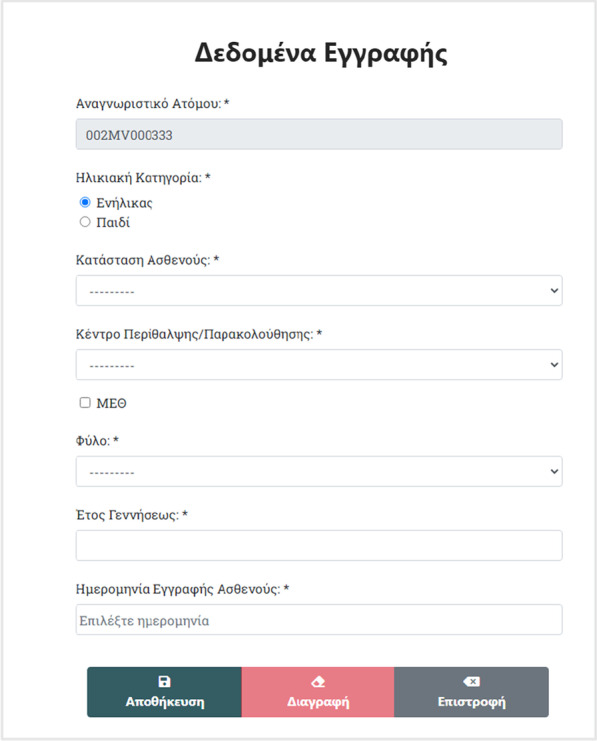
Patient’s record creation web form, asking for basic information (gender, year of birth etc.)

Patient information stored in the HR-HMV database, concerns demographic, anthropometric as well as follow-up data. Mandatory variables for each entry include information on age at diagnosis, gender, subscription date (i.e., first visit at the hospital department), region of residence, professional and educational status (in the case of a child patient these data refer to the parent/legal guardian) (Fig. 2)^5^.

**Fig. 3:**
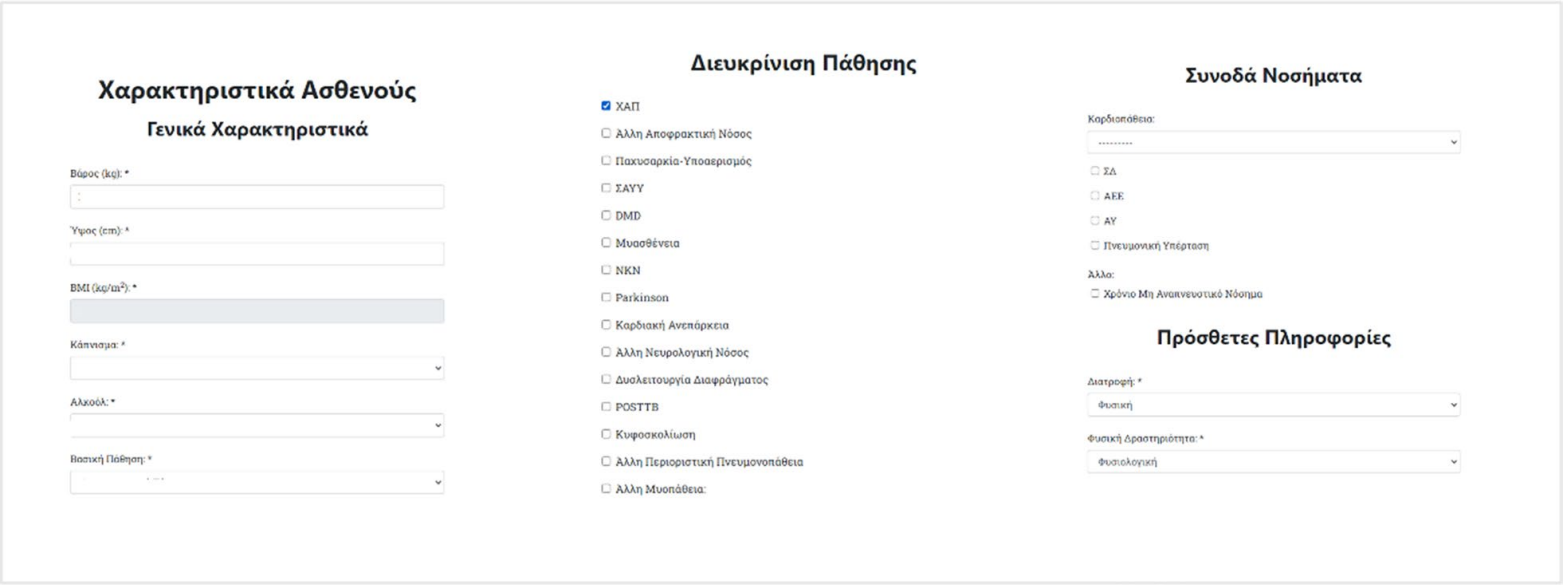
Web form asking for details for the specific patient (weight, height, smoking, regular alcohol consumption, main diagnosis, comorbidities, lifestyle habits etc.)

As far as follow-up data are concerned, patient condition, hospitalizations, height and weight (so that BMI can be calculated), smoking and alcohol habits, underlying medical conditions, nutrition and physical activity, dates and causes of potential death, as well as patient comorbidities are collected (Fig. 3).

**Fig. 4:**
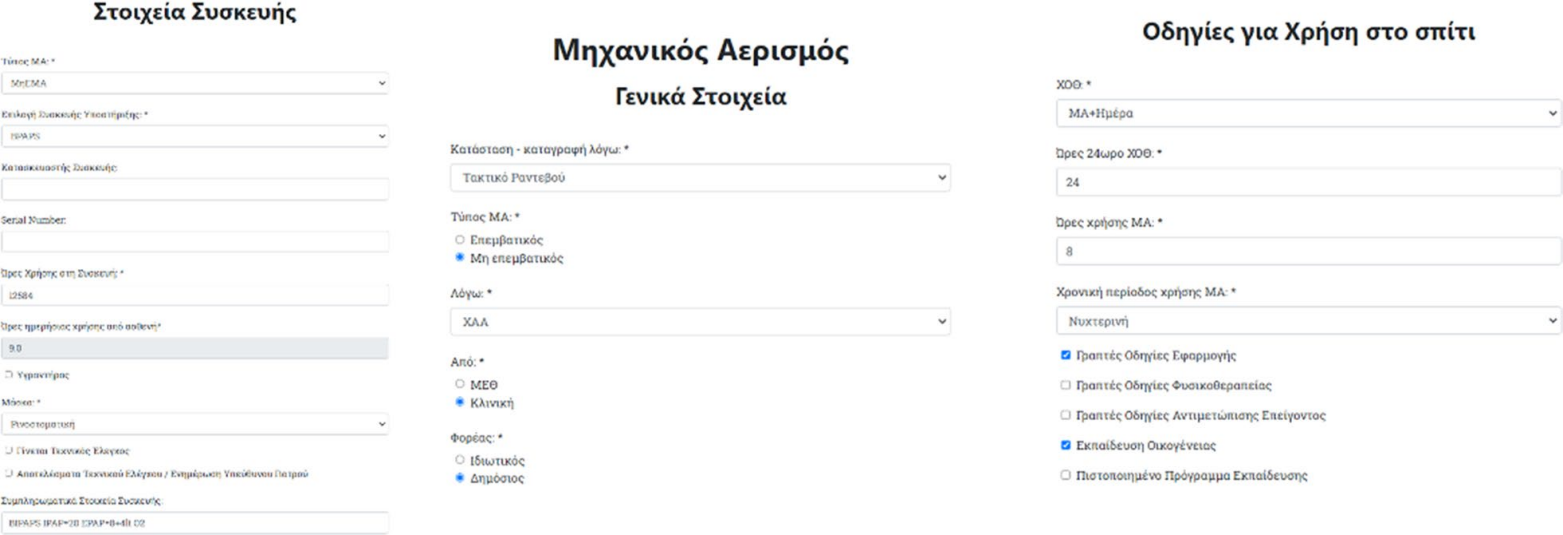
Web form asking for mechanical ventilation/PAP type

Regarding mechanical ventilation (MV), some of the most important required fields are; the reason of the visit/referral, MV usage cause, MV type (i.e., invasive, non-invasive) and the MV prescription provider. The database also includes data which refer to the use of other device at home such as additional oxygen therapy, usage hours and period of usage for the mechanical ventilation, as well as information regarding treatment instructions given, family training or certified training programs that might have been attended, concerning mechanical ventilation application (Fig. 4).

**Fig. 5:**
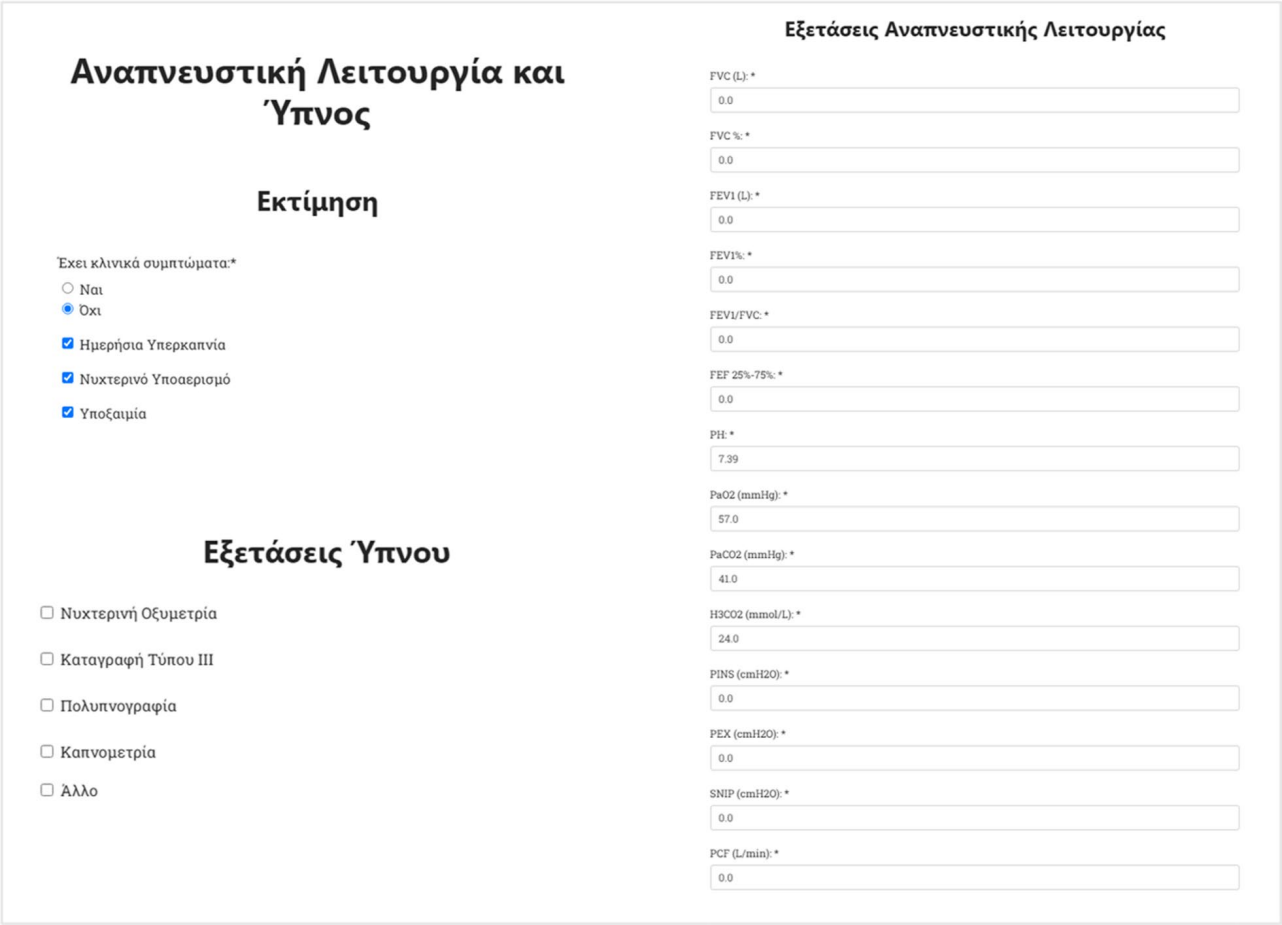
Web form asking for sleep clinical features and respiratory test results

Both baseline and follow-up laboratory tests (respiratory function and sleep tests) are stored in the HR-HMV database, as well as clinical symptoms. Additionally, information regarding the device, such as its type, usage hours, mask type used, manufacturing and maintenance details are also stored (Fig. 5).

**Fig. 6:**
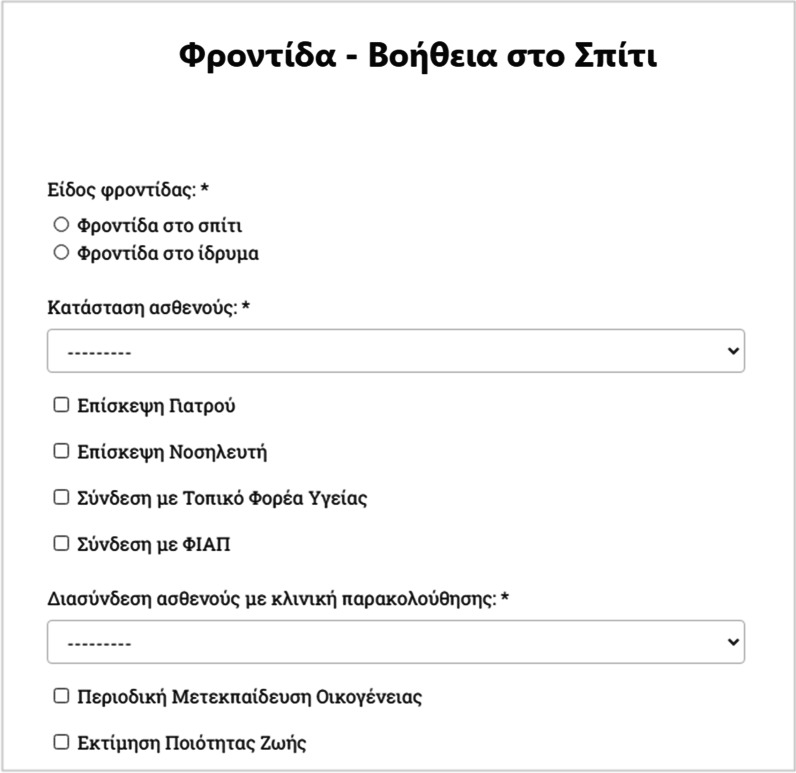
Web form used to record details for support from social services—program “Help at Home” (interconnection with specific clinic, hospitalization or home care etc.)

Finally, data depicting the condition of the patient, as well as the type and quality of care and treatment the patient receives (e.g., if it is home-care or the patient is in a healthcare organization), have been considered as vital by the HR-HMV network, and thus recorded (Fig. 6).

### Descriptive statistics

The number of patients registered at the time of writing is 5768. After cleaning data records with significant data gaps and/or erroneous values, finally, the dataset comprised 5167 patients. The registration system refers to 3 patient categories/conditions; Tracheostomy, Mechanical Ventilation and Sleep Apnea Hypopnea Syndrome which is the condition of main interest in the current study.

**Fig. 7:**
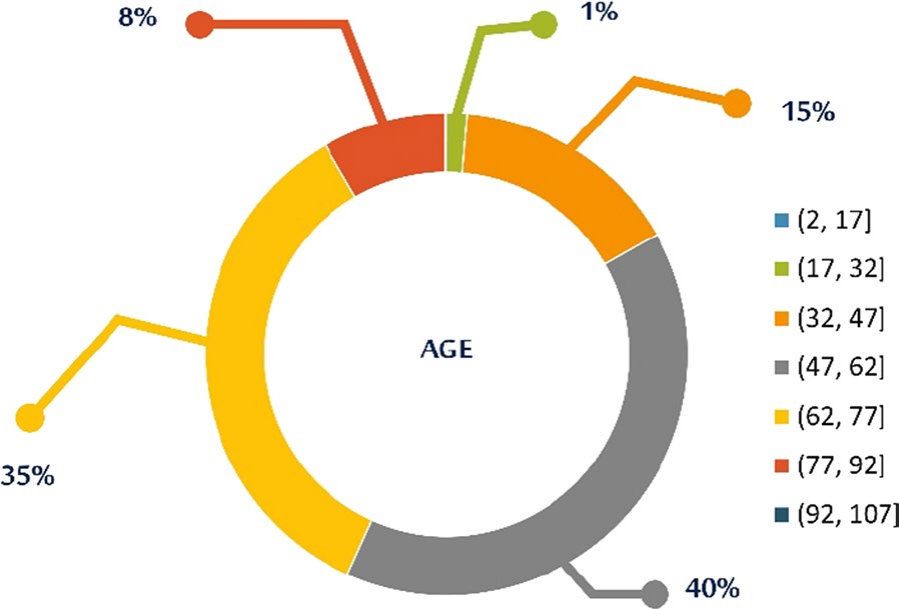
Age distribution of SAHS patients’

**Fig. 8:**
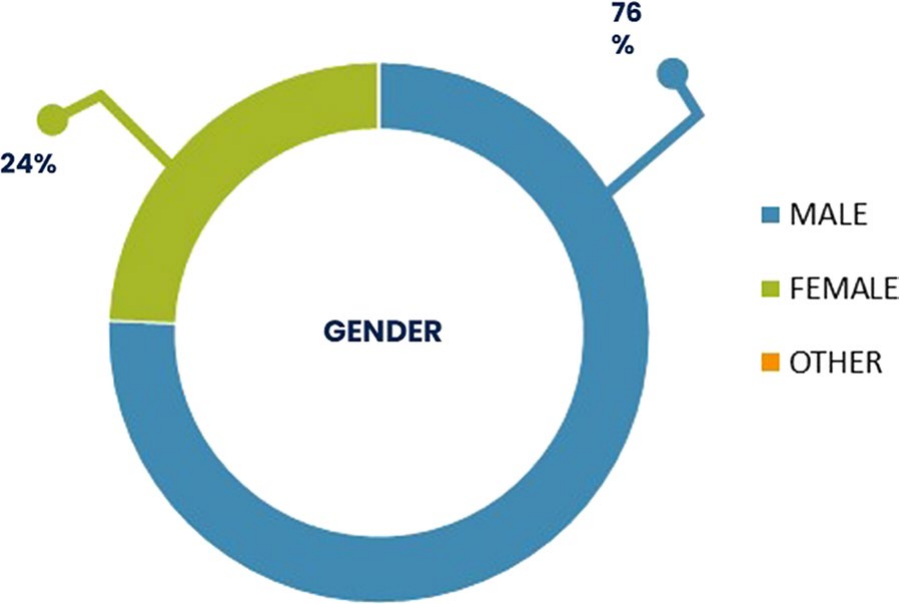
Gender distribution of SAHS patients

Focusing on the most prominent patients’ group, namely the SAHS patients (n = 4916), the median age cases was 60 years, 3,722 were male (Figs. 7 and 8).

**Fig. 9:**
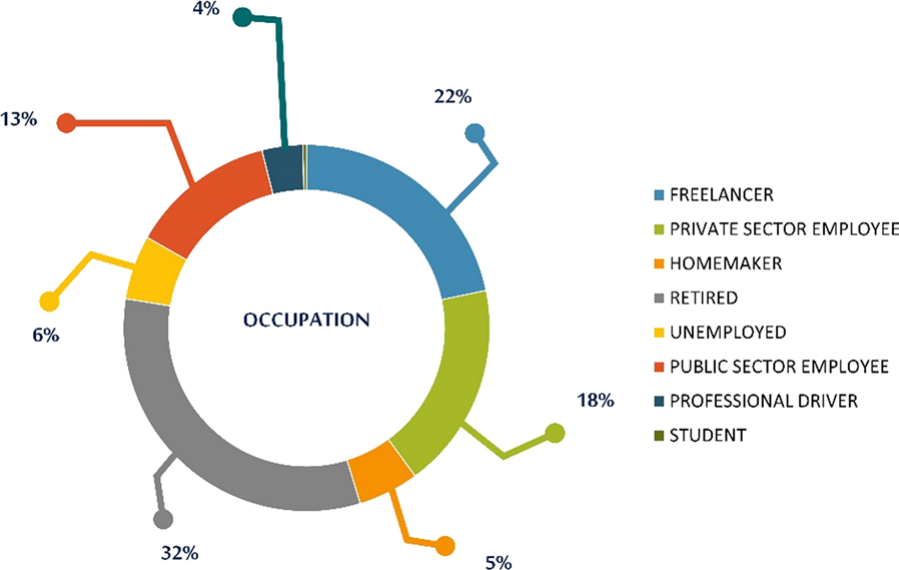
Occupational status of SAHS patients

Most of SAHS patients (Fig. 9) were people under retirement (1550), followed by freelancers (1042) and private sector employees (874).

**Fig. 10:**
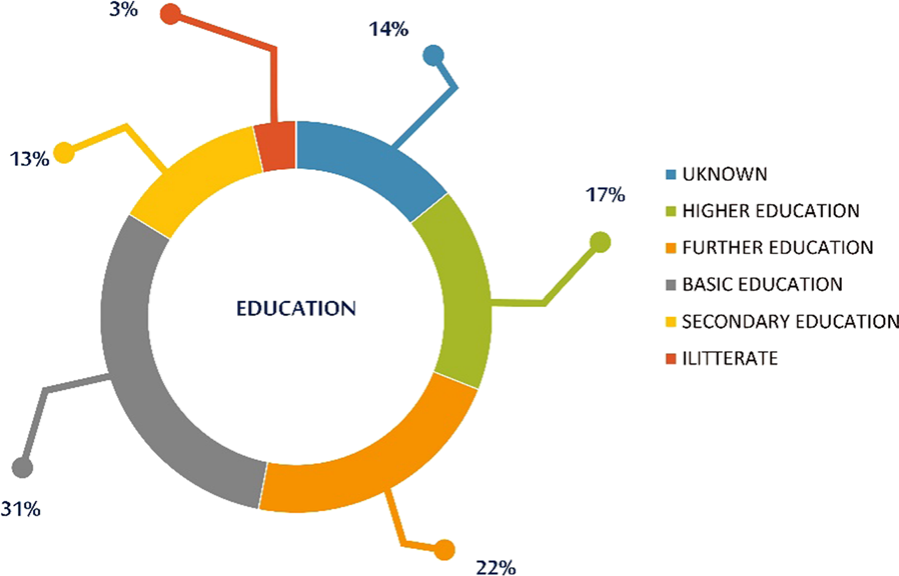
Education status of SAHS patients

The majority of the SAHS patients have received only a basic education (1473), while 22% (1059) have received further education and 17% (812) a higher education (Fig. 10). Notably, the education level of 14% (677) of patients was not recorded.

**Fig. 11:**
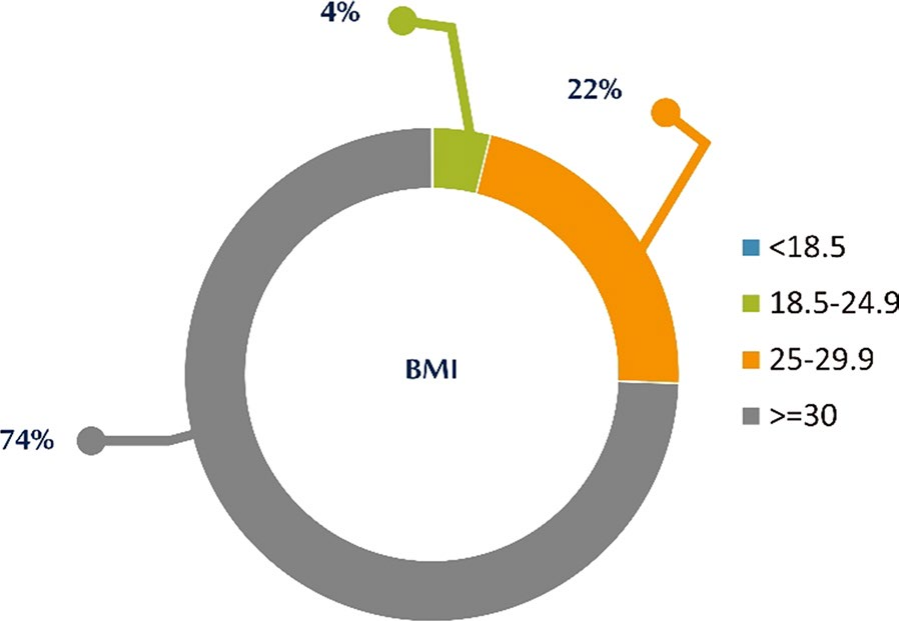
BMI distribution of SAHS patients’

The mean BMI for SAHS patients as shown by the data processing in the HR-HMV registry (Fig. 11) is 34.86 (34.33 for men and 36.51 for women).

**Fig. 12:**
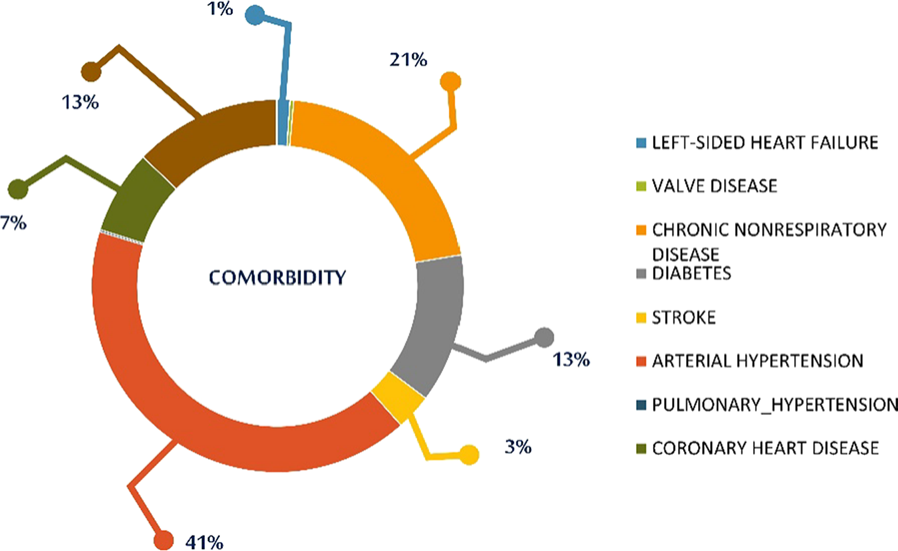
SAHS patients' comorbidities

As far as comorbidities are concerned, the most commonly observed, was arterial hypertension (2339), followed by chronic non-respiratory disease (1186) and diabetes mellitus (732) (Fig. 12).

**Fig. 13:**
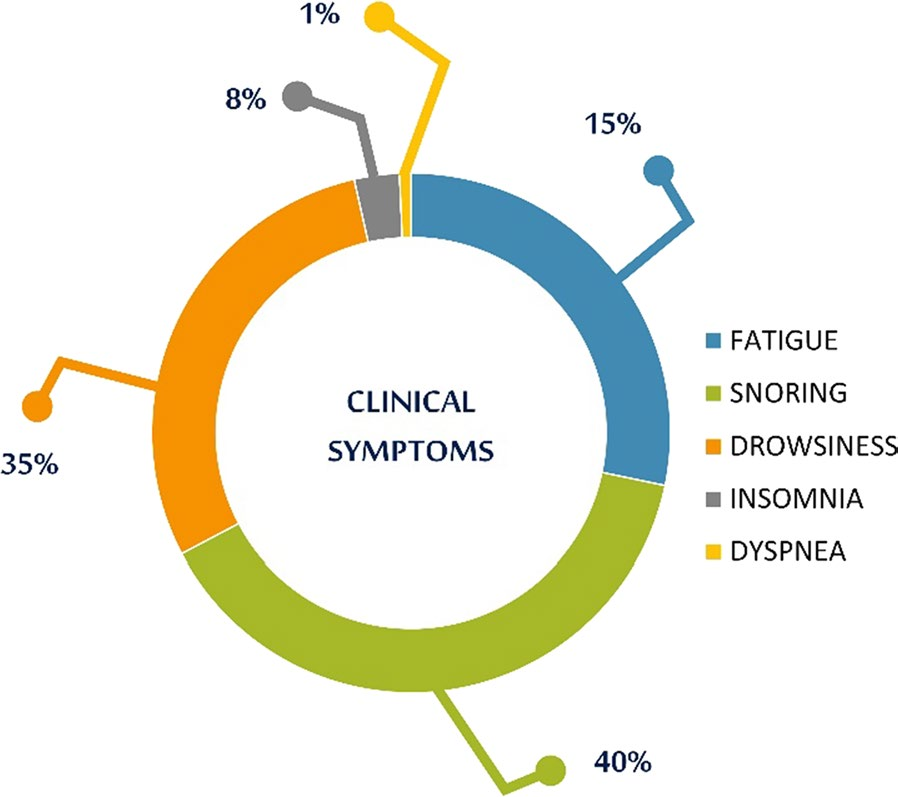
SAHS patients' clinical symptoms

Among SAHS patients, 3136 of them have reported fatigue as a clinical symptom, 4324 snoring, 3239 drowsiness, 311 insomnia and 80 dyspnea (Fig. 13).

**Fig. 14:**
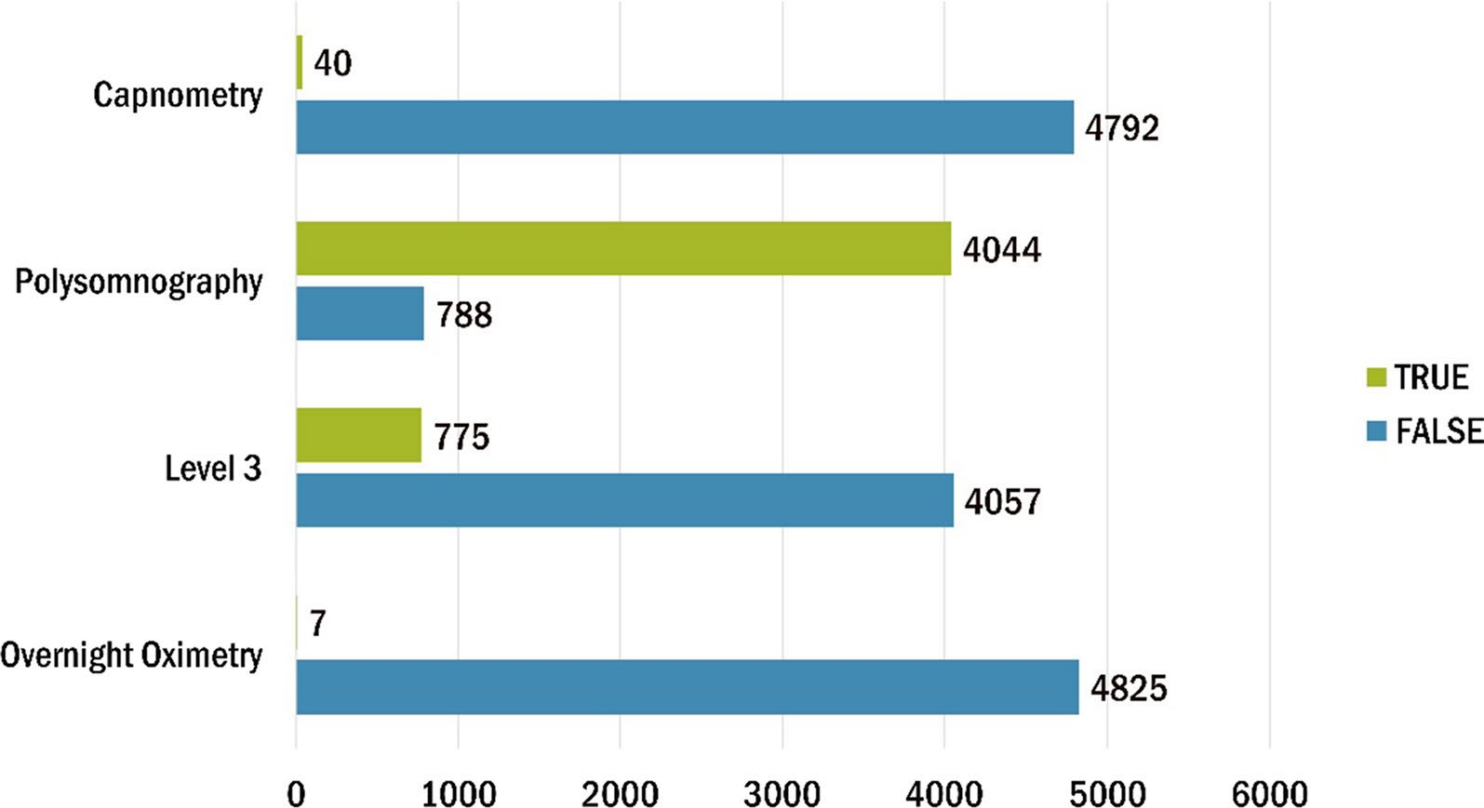
Diagnostic test for SAHS patients'

Regarding SAHS patients’ first visit, by the time of the current study, 7 patients have been subjected to overnight oximetry test, 40 to capnometry, 4044 to polysomnography and 775 to level 3 sleep study (Fig. 14).

**Fig. 15:**
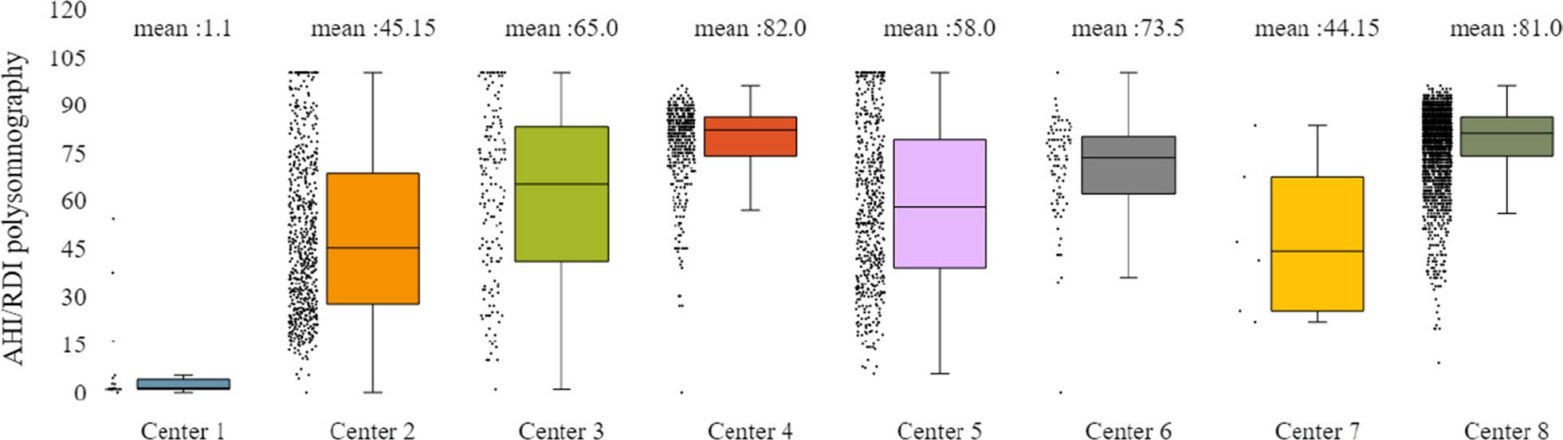
SAHS patients’ distribution with regard to the mean—std of AHI/RDI polysomnography test values, aggregating all participating hospital departments

**Fig. 16:**
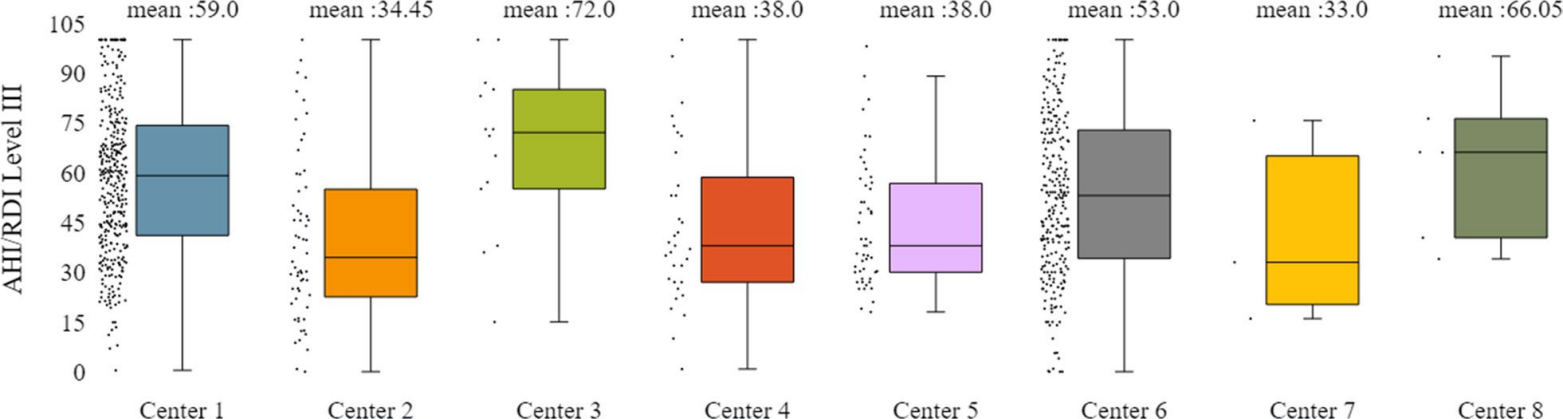
SAHS patients’ distribution with regard to the mean—std of AHI/RDI level III (polygraphy) sleep study values, aggregating all participating hospital departments

**Fig. 17:**
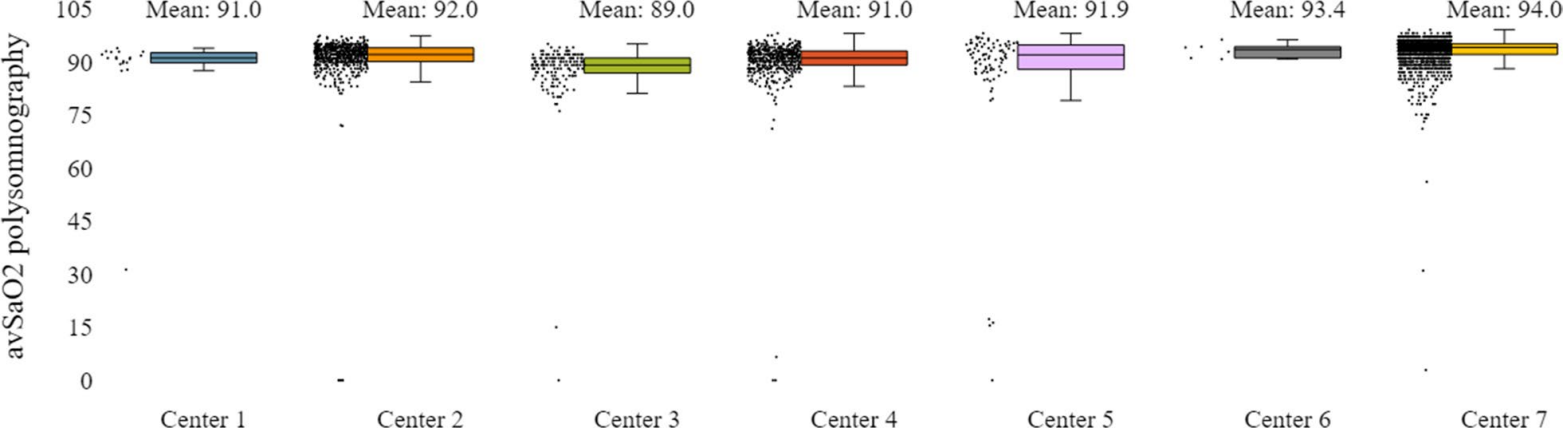
SAHS patients’ distribution with regard to the mean—std of avSaO2 polysomnography test values, aggregating all participating hospital departments

**Fig. 18:**
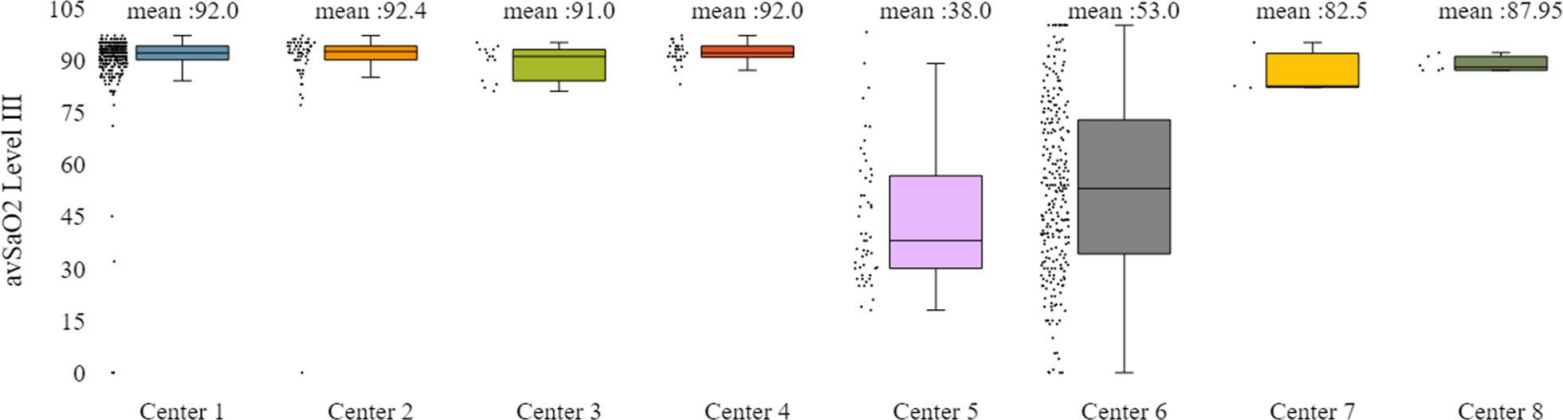
SAHS patients’ distribution with regard to the mean—std of avSaO2 level III (polygraphy) sleep study values, aggregating all participating hospital departments

**Fig. 19:**
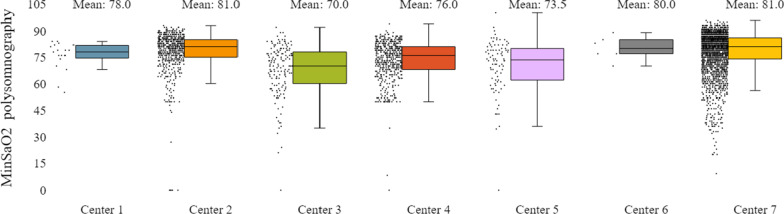
SAHS patients’ distribution with regard to the mean—std of minSaO2 polysomnography test values, aggregating all participating hospital departments

**Fig. 20:**
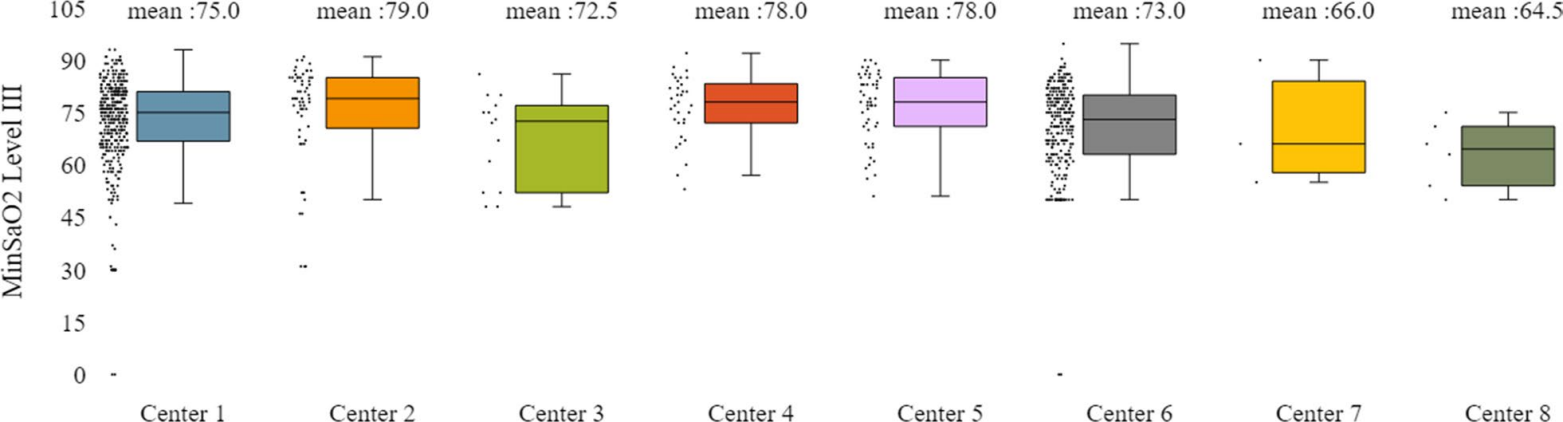
SAHS patients’ distribution with regard to the mean—std of MinSaO2 level III sleep study values, aggregating all participating hospital departments

**Fig. 21:**
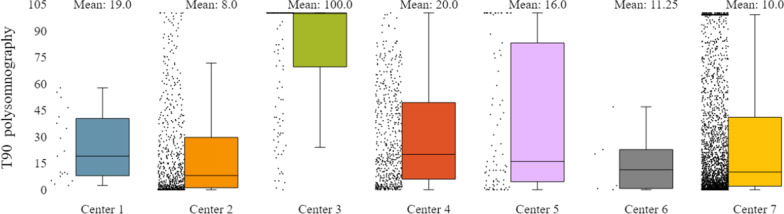
SAHS patients’ distribution with regard to the mean—std of T90 polysomnography test values, aggregating all participating hospital departments

**Fig. 22:**
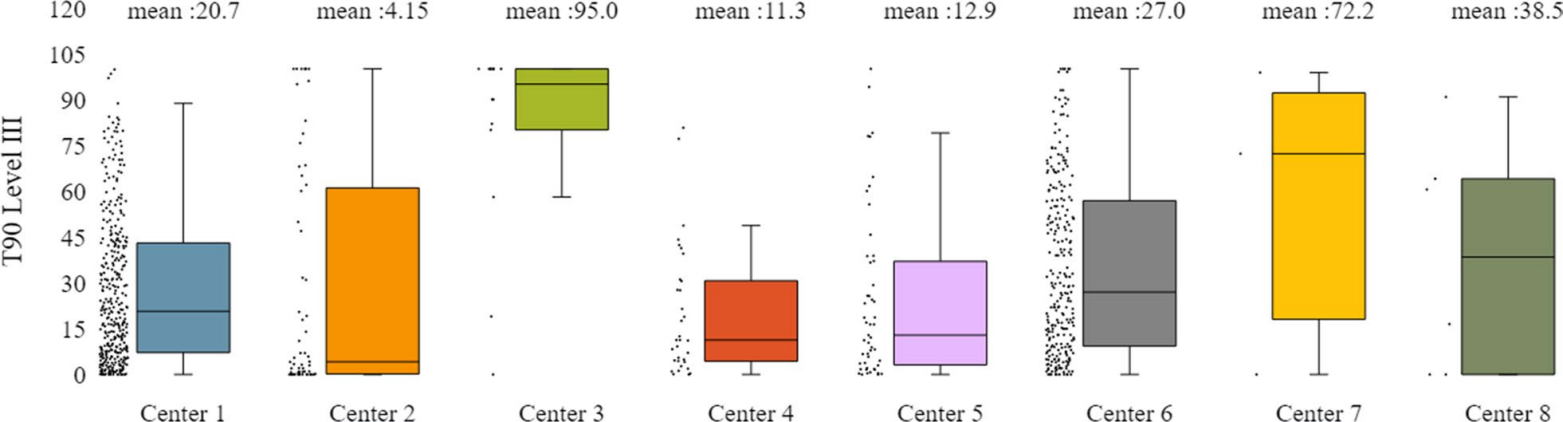
SAHS patients’ distribution with regard to the mean—std of T90 level III sleep study values, aggregating all participating hospital departments

In addition to the already mentioned general characteristics, the system allows us to have a more detailed overview of patients’ monitoring, by providing us information for specific indicators for the various tests of interest (e.g. polysomnography, level III sleep study), and sleep parameters such as AHI/RDI (Figs. 15, 16), avSaO2 (Figs. 17, 18), MinSaO2 (Figs. 19, 20) and T90 (Figs. 21, 22). These figures depict the mean and the standard deviation (std) of each metric for each of the clinical centres providing data to the HR-HMV registry. Based on these data, an outline of the respective patient population is presented highlighting some key characteristics and their distribution in the country.

## Discussion

Patient registries focusing on specific conditions have been identified as a useful tool for epidemiological research and policy making. Several initiatives have taken place aiming to deploy registries on regional, national or even international level [8–12].

Data collected in HR-HMV, could prove useful for statistical analysis and research for respiratory diseases and other chronic pathological conditions causing respiratory dysfunction, especially from the aspect of patients’ monitoring and treatment in Greece. Information contained in the registry, concerning clinical symptoms combinations, sleep tests as well as demographic and clinical characteristics of patients, could lead medical scientists to valuable conclusions and potential correlations between some of the various corresponding variables.

Additionally, as the data increase over the years, HR-HMV will probably contribute to a better understanding of the health burden of SAHS phenotypes and its association with comorbidities. A more personalized approach and treatment as well as better financial management regarding the use of ventilation devices, could be additional benefits due to the increasing collection of relevant information.

Finally, we argue that shaping and maintaining patient registries on a national level could play an important role in providing an EU infrastructure for observational research. While relevant initiatives are already in place (e.g. the EHDEN network^6^), they need to be fed by regional/national patient registries to support research but also policy or regulatory decision making processes.

## Limitations

As data quality is identified as a top priority for a registry and the data presented were not retrieved via EHR systems, the data entry process was early identified as a potential weakness of the overall process. The data were manually typed by healthcare professionals who were not dedicated to this task and therefore the data entry was executed in a batch mode. In order to mitigate the risk of bias or erroneous data entry, the platform’s user interface was designed as simple as possible, and specific guidelines were provided to data curators while integrity rules were also enforced to prevent errors.

However, systematic biases to the collected data cannot be ruled out and as these could have significant impact in the produced conclusions they should be thoroughly investigated. We consider the biased registration of patients (for example due to the selection of the contributing hospital departments) as the first and most important potential limitation for the provided data analysis and its interpretation as we did not have any means to compare our produced statistics with another data source on a national level.

Finally, regarding technical issues, the current lack of a specific coding system could be identified as a limitation. However, SNOMED-CT is going to be adopted as part of the next technical database update in order to increase the collected data semantic interoperability and facilitate their potential linking with other databases to support future studies.

## Conclusions

The HR-HMV platform is the outcome of a collaborative effort by a network of clinical units across Greece and INAB|CERTH which provides the technical expertise in terms of software engineering and, hosting and maintenance. The HR-HMV platform can be used as a data source enabling insights for the management of patients with home mechanical ventilation support, providing a valuable tool for policy decision making, observational and epidemiological research used nationwide (or even in a EU scale), already holding records for more than 5000 patients. However, both its strengths and limitations must be considered when interpreting research results, and continuous validation of its clinical data is identified as a key priority.

## Data Availability

The data that support the findings of this study are available from INAB|CERTH but restrictions apply to the availability of these data, which were used under license for the current study, and so are not publicly available. Data are however available from the authors upon reasonable request and with permission of HR-HMV network coordination committee. In case of interest please contact Dr. Pantelis Natsiavas (pnatsiavas@certh.gr).

## Footnotes

1 https://vizhub.healthdata.org/gbd-compare/.

2 https://www.who.int/data/gho/indicator-metadata-registry/imr-details/158.

3 http://www.healthdata.org/greece.

4 The data could be available for research purposes upon reasonable request.

5 The web form figures are in Greek language as the web interface aims only at Greek speaking healthcare professionals.

6 http://ehden.eu.
